# Natural language processing and machine learning to assist radiation oncology incident learning

**DOI:** 10.1002/acm2.13437

**Published:** 2021-10-05

**Authors:** Felix Mathew, Hui Wang, Logan Montgomery, John Kildea

**Affiliations:** ^1^ Medical Physics Unit McGill University Montreal Quebec H4A3J1 Canada; ^2^ Unaffiliated Montreal Quebec Canada

**Keywords:** incident learning system, natural language processing, radiotherapy incident learning, SaILS, supervised machine learning

## Abstract

**Purpose:**

To develop a Natural Language Processing (NLP) and Machine Learning (ML) pipeline that can be integrated into an Incident Learning System (ILS) to assist radiation oncology incident learning by semi‐automating incident classification. Our goal was to develop ML models that can generate label recommendations, arranged according to their likelihoods, for three data elements in Canadian NSIR‐RT taxonomy.

**Methods:**

Over 6000 incident reports were gathered from the Canadian national ILS as well as our local ILS database. Incident descriptions from these reports were processed using various NLP techniques. The processed data with the expert‐generated labels were used to train and evaluate over 500 multi‐output ML algorithms. The top three models were identified and tuned for each of three different taxonomy data elements, namely: (1) process step where the incident occurred, (2) problem type of the incident and (3) the contributing factors of the incident. The best‐performing model after tuning was identified for each data element and tested on unseen data.

**Results:**

The MultiOutputRegressor extended Linear SVR models performed best on the three data elements. On testing, our models ranked the most appropriate label 1.48 ± 0.03, 1.73 ± 0.05 and 2.66 ± 0.08 for process‐step, problem‐type and contributing factors respectively.

**Conclusions:**

We developed NLP‐ML models that can perform incident classification. These models will be integrated into our ILS to generate a drop‐down menu. This semi‐automated feature has the potential to improve the usability, accuracy and efficiency of our radiation oncology ILS.

## 1 INTRODUCTION

Radiotherapy demands coordinated involvement of a range of health professionals to manage the complexities and procedural intricacies of accurate and timely radiation treatment. Although the associated risk of misadministration is estimated to be rare, the consequences of errors may be significant.^1^, ^2^ Accordingly, the radiation oncology community has invested in incident learning systems (ILSes) to improve the safety culture, reduce incident recurrence, and prevent new incidents altogether.^3^, ^4^ Incident learning refers to the complete process of reporting and analyzing “incidents,” “near‐misses,” and “reportable circumstances” and putting in place interventions to achieve these goals.^2^

While the use of incident learning in other industries, such as aviation and nuclear power, is well established,^5^, ^6^ its application in healthcare is relatively new. In radiotherapy, various ILSes came into existence only in the last two decades or so^7^, ^8^, ^9^ and are in regular use today at institutional, regional, and national levels. In Canada, the National System for Incident Reporting—Radiation Treatment (NSIR‐RT)^10^ is an incident classification taxonomy and the associated national ILS was implemented in 2015 in an effort to standardize Canadian radiotherapy incident learning practices. NSIR‐RT was developed by the Canadian Partnership for Quality Radiotherapy^11^ and is managed by the Canadian Institute for Health Information (CIHI).^12^ NSIR‐RT has been adopted by almost all Canadian radiotherapy centers and its taxonomy has been integrated into several commercial and open‐source ILS software. In 2016, our group incorporated the NSIR‐RT taxonomy into an open‐source ILS software called the Safety and Incident Learning System (SaILS)^13^, ^14^ and deployed it in our radiotherapy center as part of a quality and safety improvement project.^15^

As shown in Figure 1a, the SaILS incident reporting interface includes only a small number of data elements, in order to facilitate rapid submission of incidents into the database. The most substantial component of the initial incident report is the incident description, which is a free‐text description of the incident that should be written in a no‐blame manner. Each reported incident is assigned an investigator, who is alerted by email to complete the incident classification using SaILS’ investigation interface, shown in Figure 1b. To do so, the investigator must classify the incident by selecting the most appropriate label(s) from a dropdown list for each NSIR‐RT data element.

FIGURE 1(a) Screenshot of the reporting interface of the Safety and Incident System (SaILS) as seen by an incident reporter (left), (b) screenshot of the SaILS investigation interface (right); figures obtained from Montgomery et al.^15^

**Figure:**
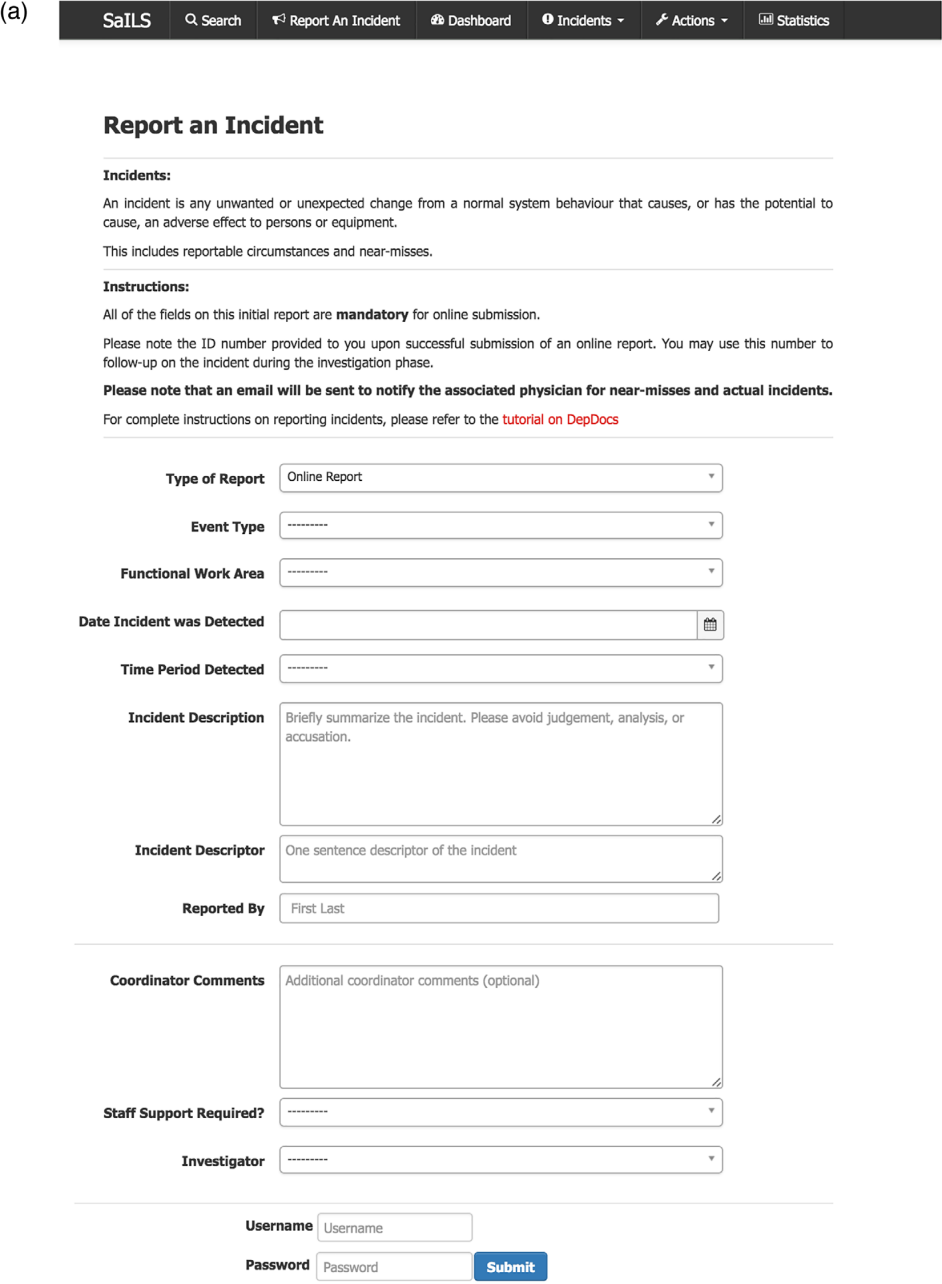

**Figure:**
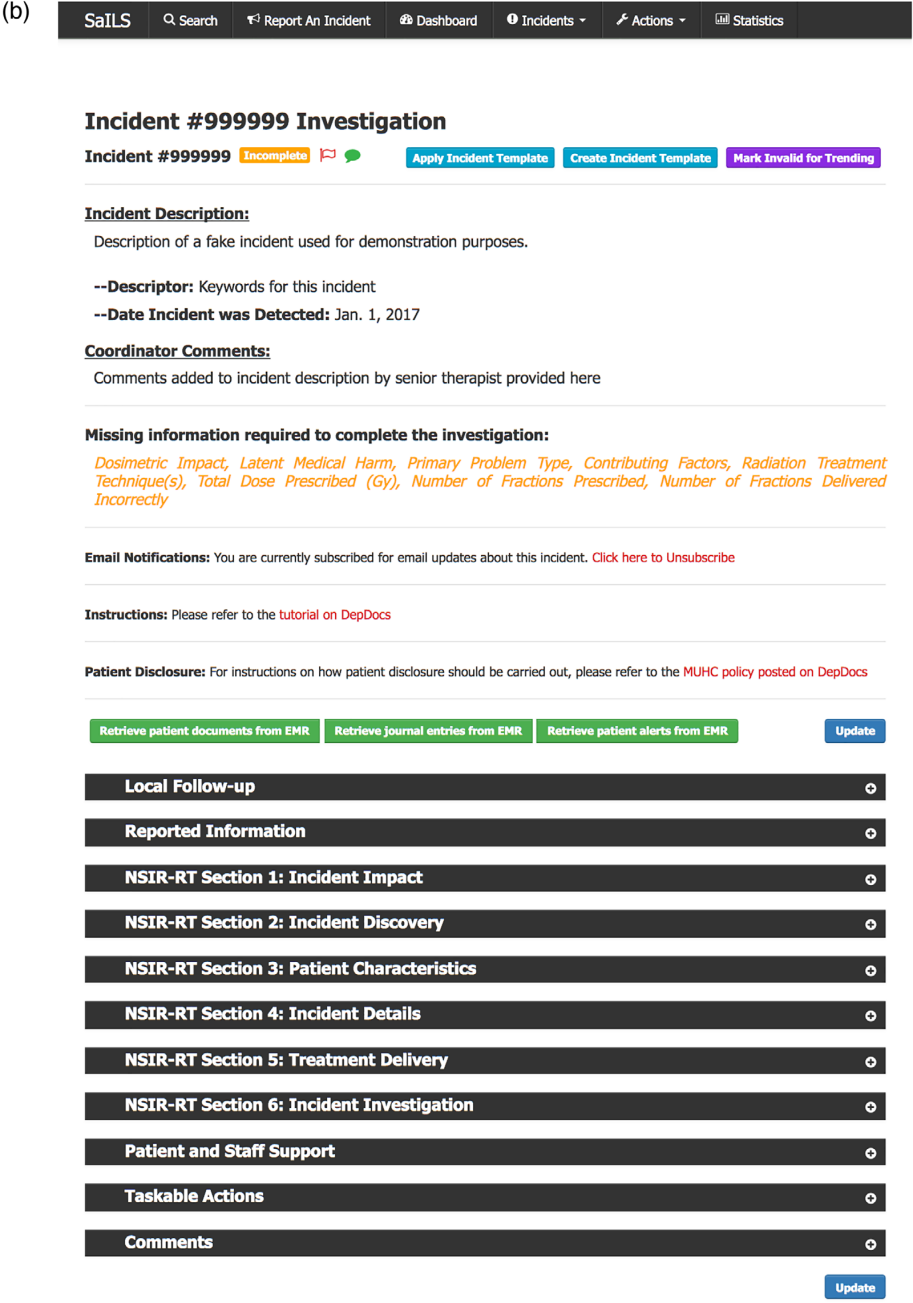

A total of 1587 incidents were reported in SaILS between January 2016 and December 2020, yielding an average reporting rate of about 26 incidents per month for our radiotherapy center, which treats approximately 325 new patients per month. Analyzing, investigating, and labelling these incidents manually is an arduous task that requires dedicated time and resources. Manual classification of an incident can be difficult, especially because the most important information is described as free text in the incident report. This motivated us to attempt automated classification by converting unstructured data (free text) into structured information using natural language processing (NLP)^16^ and machine learning (ML).^17^

**FIGURE 2:**
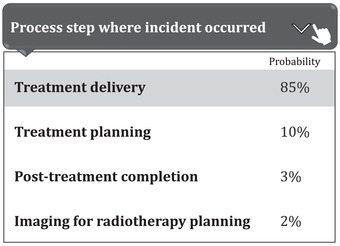
A mockup of the drop‐down menu that lists the “process step where the incident occurred” label recommendations in the order of their probability, according to a machine learning model applied to the corresponding incident description following natural language processing; each label recommendation and its associated probability of being appropriate for the incident under investigation is shown

Supervised ML methods^18^, ^19^ that aim to predict the classification labels (class labels) of new incident reports by learning from expert‐labelled training data have been used previously in the field of medical incident learning.^20^, ^21^, ^22^ However, it is acknowledged that ML approaches cannot yet replace manual incident classification^21^, ^23^, ^24^ due to their imperfections and inaccuracies. For this reason, the goal of our study was to build ML models that can assist the investigator rather than attempt to fully automate incident classification in radiation oncology. Our intention was to develop models that can learn from previously labelled incident reports and generate a dropdown menu that displays suggested class labels for each new incident in descending order of probability of being correct. A mockup of the data element dropdown menu for the “process step where the incident occurred” is shown in Figure 2. With this approach, investigators can read the incident description as normal but with our dropdown menu, they can save time during the label selection. Our models are intended to make the investigation process more convenient and to serve as a safety net for the investigators.

In this manuscript, we describe how we developed supervised NLP‐ML models that can generate label recommendations for three data elements in the NSIR‐RT taxonomy. The three data elements are (i) the process step where the incident occurred, (ii) the type of problem reported, and (iii) the contributing factors that led to the incident. These data elements have 8, 16, and 25 possible labels, respectively. We chose these three data elements for this study because their labels are typically derived directly from the incident descriptions, without requiring further investigation.

## 2 METHODS AND MATERIALS

### 2.1 Data sources

**FIGURE 3:**
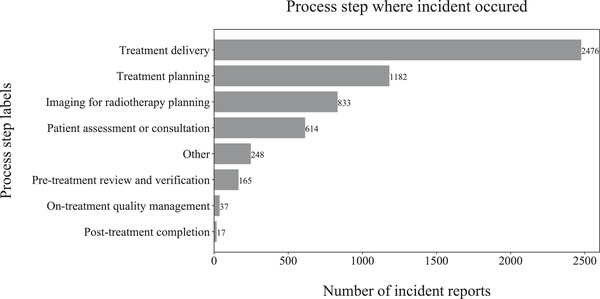
Histogram showing the process step label distribution in our dataset of 5572 radiation oncology incident reports; out of the eight label options available, each report was labelled with one process step

**FIGURE 4:**
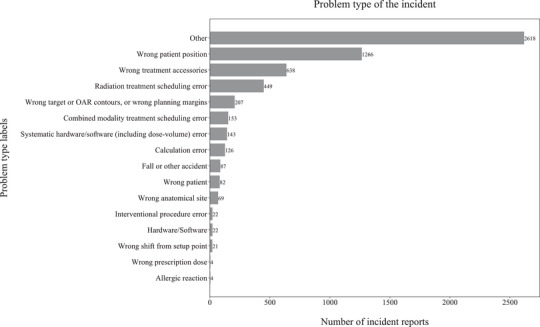
Histogram showing the problem type label distribution in our dataset of 5911 labelled radiation oncology incident reports; out of 16 label options available, each report was labelled with one problem type

**FIGURE 5:**
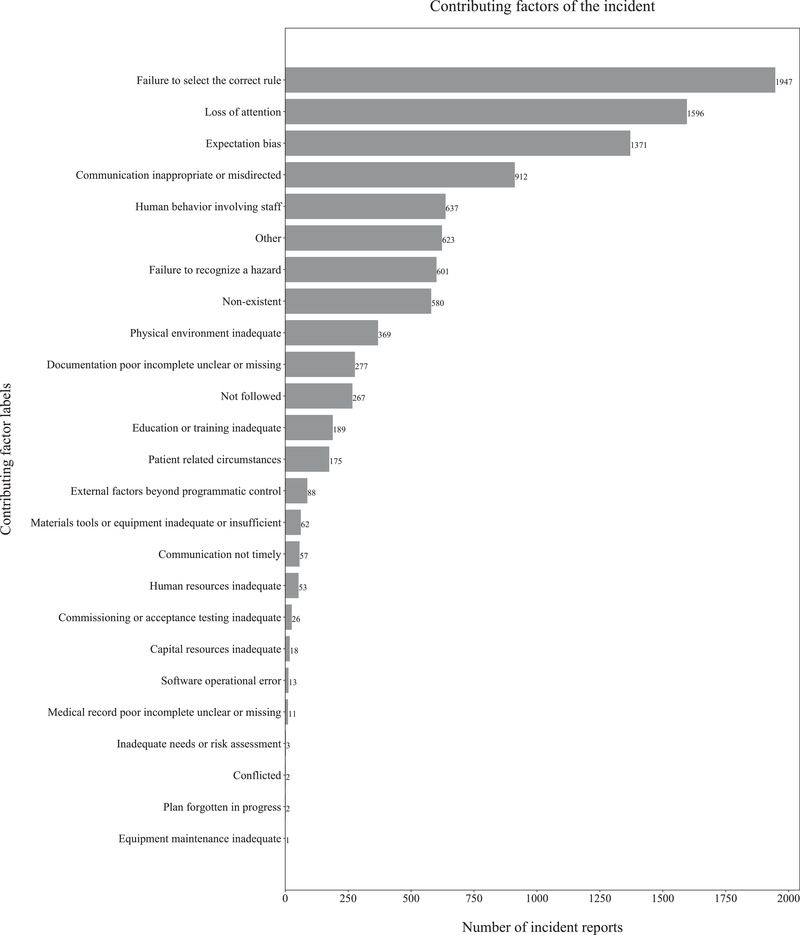
Histogram showing the contributing factor label distribution in our dataset of 5909 labelled radiation oncology incident reports; out of 25 label options available, each report was labelled on average with two contributing factors

We obtained 1587 expert‐labelled radiation oncology incident reports from our SaILS database and an additional 5098 incidents from the national NSIR‐RT database managed by CIHI. SaILS uses the 2015 pre‐pilot version^25^ of the NSIR‐RT taxonomy whereas CIHI uses the 2017 post‐pilot version,^26^ which introduced new class labels and modified some pre‐existing ones. Therefore, we consolidated the data by mapping the CIHI data to the pre‐pilot taxonomy, following the CIHI guidelines (Spencer Ross, CIHI, email communication, 25 February 2020), in order to build a model that works in SaILS. Because several of the incidents were still under investigation (i.e., incomplete) when the data were collected, not all data elements were labelled. Thus, we had a total of 5572, 5911, and 5909 reported incidents with expert‐generated labels corresponding respectively to the process step, problem type, and contributing factors data elements. The inherently imbalanced distribution of class labels for these three data elements in our dataset is shown in Figure 3, Figure 4, and Figure 5, respectively. The reason for this imbalance relates to the nature of radiotherapy delivery and the inherent probabilities for incidents to occur. Note that only a single label may be assigned for the process step and problem type data elements, while multiple labels may be assigned for the contributing factors data element.

### 2.2 NLP of the incident descriptions

The CIHI system is bilingual and hence our dataset consisted of incident descriptions in both French and English. Additionally, these descriptions contained grammatical errors, spelling mistakes, improper sentence structure, and shorthand that needed to be processed to improve the performance of the ML models. In this section, we describe how we used various NLP techniques to process the free‐text incident descriptions to render them suitable for input into ML models.

This study was performed entirely using various Python packages (Python 3.8). The text processing techniques that we implemented and the corresponding Python packages were:

1. Line‐break removal: New lines or line breaks (“∖n” characters) were replaced by periods (full stops) to introduce breaks after complete sentences using the built‐in “replace” function in Python.
2. Translation: We used the Google translate API package in Python called “google‐trans‐new” version 1.1.9^27^ to translate French incident descriptions into English. Using the language detection function, we identified the reports that were in French and then translated them.
3. Punctuation and whitespace removal: Python's standard library for regular expression operations^28^ was used to remove all the punctuation marks and unnecessary blank spaces between words in every sentence.
4. Lowercase normalization: In this step, all letters were transformed to their lowercase equivalent to ensure that instances of the same word that were written differently were identified as a single object (e.g., Radiation, radiation, RADIATION, etc.).
5. Autocorrection: Using Python's spell‐checking package “PyEnchant” version 3.1.1,^29^ all words that had spelling errors according to the US English PyEnchant dictionary were identified and corrected. PyEnchant falsely identifies some radiation oncology terms as incorrect, such as “vacloc,” “brachy,” and “isoshift.” These were manually identified and exempted from autocorrection.
6. Entity replacement: The advanced NLP package SpaCy (version 2.3.2)^30^, ^31^ has a textual entity recognition feature. This feature was used to identify words or ordinals that describe time, date, quantity, and percentage in our text and replace them with a generic label that describes the entity. For example, knowing an exact date (e.g., 2 June 2020) holds no more significance than knowing that entity is a “date” in the context of ML classification.
7. Stopword removal and lemmatization: Stopwords are words that are used frequently in English, such as “the,” “an,” “and,” “with,” and “but.” These words have no classification value and can degrade text classification in some scenarios.^32^ We used SpaCy to remove all stopwords and subsequently applied its lemmatization feature to normalize the text. Lemmatization refers to the process of replacing words with their basic dictionary form (lemma).^33^ For instance, the words “working,” “works,” and “worked” were changed to their normalized form “work.”

Table 1 in results shows some examples of how these techniques transformed the radiation oncology incident descriptions.

**TABLE 1:** Five example incident descriptions from our dataset are shown in their original format as well as after processing in our natural language processing pipeline for comparison. The processed text demonstrates the impact of lowercase normalization, autocorrection, punctuation and whitespace removal, entity replacement, stopword removal, and lemmatization

Example no.	Original incident description	Processed text
1	INCCURATE TARGET. CTV WAS BIGGER THAN PTV, WAS NOTICED ONLY AT THE END OF PLANNING PROCESS, TARGET HAD TO BE CORRECTED AND PLAN REDONE.	Inaccurate target ctv big ptv notice end planning process target correct plan redone
2	No review status . Pt started rt on May 22, films were never reviewed until May 27th. Reached 7/9 and MD never verified films	Review status pt start rt date film review date reach md verify film
3	No anti‐emetic. 1 shot spine plus 25/5 rectum. Anti‐emetic never prescribed for spine treatment. Danger of patient being sick during rectum iso and treat.	Anti‐emetic shoot spine plus quantity rectum anti emetic prescribe spine treatment danger patient sick rectum iso treat
4	Not enough time to do the work, risk for mistakes. Waiting time for patient. PLAN 2 received last minute to do the plan in dosi 2 h before the patient appointment	Time work risk mistake wait time patient plan receive time plan dosi hours patient appointment
5	Patient orientation. During treatment set up, we noticed that the documented patient orientation was wrong. Patient was scanned HEAD FIRST, but the ct‐sim set up sheet indicates FEET FIRST	Patient orientation treatment set notice document patient orientation wrong patient scan head ordinal ct sim set sheet indicate foot ordinal

### 2.3 Supervised learning

In this section, we describe the steps involved in developing ML models that can generate ranked lists of appropriate class labels in order of their probability. All of the Python packages described in this section were obtained from the Scikit‐learn ML module^34^ (version 0.23.1).

#### 2.3.1 Data vectorization

The NLP‐processed incident descriptions and their associated class labels were required to be converted into vectors or matrices that the ML model could understand. The process of transforming text data into numerical arrays is known as data vectorization in NLP.

The NLP‐processed incident descriptions were vectorized by means of the Term Frequency–Inverse Document Frequency (TF‐IDF) vectorizer of Scikit‐learn. The TF‐IDF vectorizer assigned a weighted score to every term in the description based on its frequency of occurrence in the entire incident dataset when generating the matrix of features (words). This facilitated the determination of the most relevant features when categorizing a large number of reports. Individual reports were represented as rows of this multidimensional matrix. In order to conserve some of the interterm relations, we specified bigrams (e.g., “green mattress” and “plan ready”) and trigrams (e.g., “external beam plan” and “request rad onc” ) to be vectorized, keeping the word order unchanged, in addition to the individual words. Only words and bigrams with a minimum frequency of three were considered for vectorization.

The class‐label data were vectorized using the one‐hot encoding (OHE) technique.^35^ OHE generates a matrix Y_ij_, where i spans the number of reports in our dataset labelled for a given data element and j is the number of labels for that data element. Y_ij_ took the value 1 when i^th^ report was labelled with j^th^ label and in all other cases, Y_ij_ was set to zero.

#### 2.3.2 Model selection and training

Selecting the optimal ML algorithm (referred to as an estimator in Scikit‐learn) for a specific problem can be challenging, especially given a large number of available estimators. An estimator can be a classifier or a regressor algorithm. Classifiers are ML algorithms that can categorize data into discrete categories whereas regressors are algorithms that can predict continuous variable quantities. Our approach was to evaluate the performance of all available estimators on our dataset and to select the best. We obtained 52 base estimators and five estimator ensembles from the Scikit‐learn library. Ensembles are collections of base estimators that work together to generate robust models that are more generalizable.^34^ These ensembles are meta‐estimators that take another estimator as a parameter to build a model. Therefore, we obtained 260 ensemble‐base combinations in addition to the 52 base estimators to evaluate.

We were faced with a multilabel classification problem^36^ for which the goal was to generate a ranked list of possible class labels, unlike predicting a single label per incident. Scikit‐learn provides four techniques (two each for classifiers and regressors) to extend an estimator's functionality to support multilabel classification. These are the MultiOutputClassifier, ClassifierChain, MultiOutputRegressor, and RegressorChain multi‐output techniques. Among the 312 estimators compiled, classifiers used both MultiOutputClassifier and ClassifierChain methods and regressors used both MultiOutputRegressor and RegressorChain methods to enable the multilabel capability. Thus, we assembled more than 600 extended multi‐output estimators, some of which were not compatible with the technique and raised execution errors on training, which we later removed from our pipeline.

Multilabel techniques work on the principle of solving for a single label at a time, by assigning one instance of the estimator for every label option available. In practice, this implies, for a data element with 16 label options, for example, the multilabel model will generate 16 instances of an estimator and assign one for each label. These 16 instances will try to find if their label is best fitting for the incident, either in parallel (MultiOutputClassifier and MultiOutputRegressor) or in series (ClassifierChain and RegressorChain). This ability to fit the model on individual label options and to get fit scores for each label lets us also use regressors for our classification problem.

We evaluated the performance of all these multi‐output estimators on the training set (80% of the dataset, chosen randomly) using the fivefold cross‐validation technique.^37^ In K‐fold cross validation (K = 5 in our case, chosen based on the size of our training dataset), the data are split into K equally sized groups and the algorithm iteratively uses each unique group for performance evaluation while the rest are used for training until K groups are used once.

A scorer named “TrueLabelIndex scorer” was custom built using the make_scorer function of Scikit‐learn, which measured the average index of the correct class label in the ranked list, according to probability, as predicted by an estimator. For instance, if the TrueLabelIndex scorer assigns a value of 2 for an estimator, it implies that the estimator was able to place the correct label as the second‐most probable option in the ranked list, on average. In other words, if this estimator was used to build the dropdown menu in SaILS, as shown in Figure 2, investigators will see the most appropriate label appearing as the second suggestion in the dropdown list most of the time. In the case of contributing factors, where there was more than one label for every incident, the index of the contributing factor that first appeared in the predicted dropdown list was considered to be the TrueLabelIndex score. Based on this TrueLabelIndex score and the training time, we shortlisted three trained estimators (models) per data element that performed the best and tuned them, as described in the following section.

#### 2.3.3 Model tuning and final testing

**FIGURE 6:**
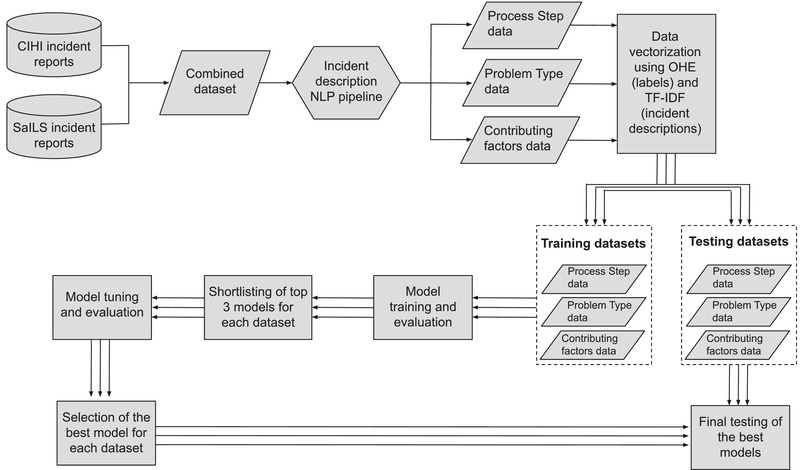
A flowchart describing the stages of our natural language processing–machine learning model development; simultaneous procedures for each data element are represented by parallel lines/arrows

Each model has a set of parameters that can take a wide range of values. Hyperparameter tuning is the process of identifying the parameter values that maximize a model's performance. We automated the hyperparameter tuning of the top three models for each taxonomy data element ((i) the process step where the incident occurred, (ii) the type of problem reported, and (iii) the contributing factors that led to the incident) using the *GridSeachCV* function of the Scikit‐learn. Grid search is essentially a trial‐and‐error approach in which the model is tested on an exhaustive set of parameters and evaluated each time a parameter is changed until the best score is attained. Of the three shortlisted models per data element, we selected a single, tuned model that performed the best for that data element. These tuned models were then tested on the test set (20% of the dataset), which the models were seeing for the first time and for which the final test score was noted. A flowchart of our complete NLP‐ML model development pipeline is shown in Figure 6.

### 2.4 Benchmarking our model

As shown in Figures 3, 4, and 5, our raw data have an inherent label distribution that is not uniform. Therefore, we undertook a benchmarking analysis to ensure that our ML model performance was superior to a baseline analytical approach. In this analytical approach, we generated another ranked list of label suggestions for each of the three data elements that we considered. These lists were generated by simply arranging the label options in order of decreasing frequency, as per the distributions shown in Figures 3, 4, and 5. This analytical approach was evaluated by applying it to our testing data set, measuring the resulting TrueLabelIndex, and comparing the results with our ML model.

## 3 RESULTS

Table 1 shows some examples of how our NLP pipeline transformed the radiation oncology incident descriptions. Example 1 in the table clearly shows the impact of lowercase normalization, punctuation and whitespace removal, autocorrection of the word “inaccurate,” stopword removal, and lemmatization of the word “bigger.” In example 2, the words “May 22” and “May 27th” are replaced with the word “date,” which is the effect of entity replacement.

Tables 2, 3, and 4 show the top three models that performed the best with respect to the TrueLabelIndex score on the training data for the process step, problem type, and contributing factors datasets respectively. We found that the MultiOutputRegressor method of enabling multilabel classification was the fastest and most accurate across all three datasets.

**TABLE 2:** TrueLabelIndex scores for the three best‐performing models that were evaluated for classification of the process step data element on the training data with fivefold cross validation; the TrueLabelIndex scorer we custom built for model evaluation scored on a scale of 1–8, for which the ideal score is 1

Models trained on process step dataset	TrueLabelIndex score (1–8; best score = 1)
MultiOutputRegressor + Ridge	1.57
MultiOutputRegressor + Linear SVR	1.71
MultiOutputRegressor + SGD Regressor	2.07

**TABLE 3:** TrueLabelIndex scores for the three best‐performing models that were evaluated for classification of the problem type data element on the training data with fivefold cross validation; the TrueLabelIndex scorer we custom built for model evaluation scored on a scale of 1–16, for which the ideal score is 1

Models trained on problem type dataset	TrueLabelIndex score (1–16; best score = 1)
MultiOutputRegressor + SGD Regressor	2.96
MultiOutputRegressor + Linear SVR	2.98
MultiOutputRegressor + Passive Aggressive Regressor	3.38

**TABLE 4:** TrueLabelIndex scores for the three best‐performing models that were evaluated for classification of the contributing factors data element on the training data with fivefold cross validation; the TrueLabelIndex scorer we custom built for model evaluation scored on a scale of 1–25, for which the ideal score is 1

Models trained on contributing factors dataset	TrueLabelIndex score (1–25; best score = 1)
MultiOutputRegressor + SGD Regressor	4.32
MultiOutputRegressor + Lasso Lars	4.88
MultiOutputRegressor + Linear SVR	7.62

**FIGURE 7:**
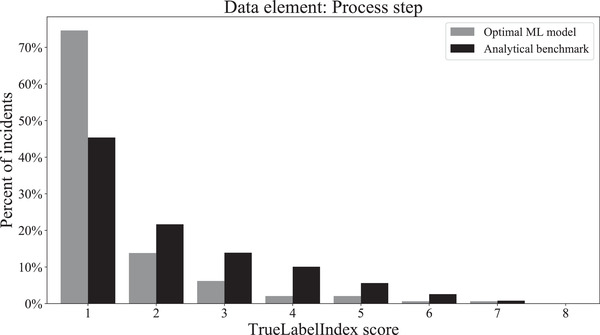
Distribution of TrueLabelIndex scores obtained for the process step dataset for the label predictions made by the optimal machine learning model and the analytical benchmark on the final test set

**FIGURE 8:**
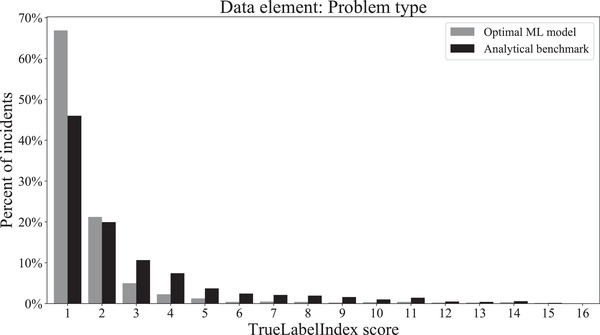
Distribution of TrueLabelIndex scores obtained for the problem type dataset for the label predictions made by the optimal machine learning model and the analytical benchmark on the final test set

**FIGURE 9:**
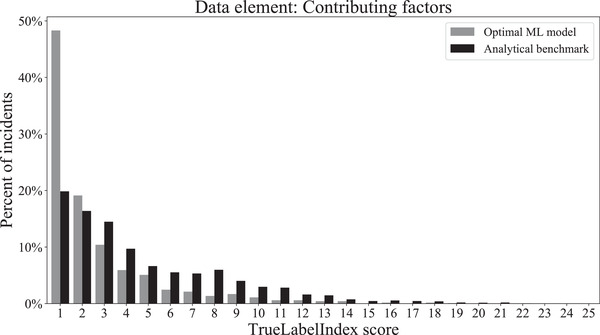
Distribution of TrueLabelIndex scores obtained for the contributing factors dataset for the label predictions made by the optimal machine learning model and the analytical benchmark on the final test set

Hyperparameter tuning of the top three models for each dataset revealed that the combination of MultiOutputRegressor with the Linear SVR (support vector regressor) base estimator was the most accurate model for our data. This finding was consistent across all three datasets corresponding to the process step, problem type, and contributing factors labels. The final TrueLabelIndex score, obtained when this model was used to predict the labels of the unseen test set, is given in Table 5. The TrueLabelIndex score obtained using the benchmarking analytical approach is also included for the purpose of comparison. The distributions of the final test scores that were obtained with the optimal ML model as well as with the benchmarking analytical model are shown for each data element in Figures 7, 8, and 9, respectively.

**TABLE 5:** The final test—TrueLabelIndex scores of the optimal, trained models for each of the three data elements, after hyperparameter tuning; uncertainties are the standard error of the corresponding mean value. The final test score was obtained by testing the model on unseen data and the closer the score is to 1, the better the model is. The score range for the process step, problem type, and contributing factors data elements were 1–8, 1–16, and 1–25, respectively. The TrueLabelIndex score from the benchmarking analytical approach is included for comparison

Data element	Optimal machine learning (ML) model	TrueLabelIndex score obtained with ML model (Best score = 1)	TrueLabelIndex score obtained with analytical approach (Best score = 1)
Process step	MultiOutputRegressor + Linear SVR	1.48 ± 0.03	2.20 ± 0.04
Problem type	MultiOutputRegressor + Linear SVR	1.73 ± 0.05	2.74 ± 0.08
Contributing factors	MultiOutputRegressor + Linear SVR	2.66 ± 0.08	4.75 ± 0.09

## 4 DISCUSSIONS

Incident learning using ML techniques is a relatively new approach in radiation oncology. To the best of our knowledge, the only published report of using NLP‐ML methods to automate radiotherapy incident learning was by Syed et al. in 2020.^38^ In their study, they used traditional ML as well as transfer learning methods to perform binary classification of incident severity (high vs. low). Therefore, our work complements the literature by providing first‐hand insight into the capability of NLP‐ML methods to classify three other radiotherapy incident data elements, namely: the process step of incident occurrence, problem type, and contributing factors. Our work had the additional advantages of a large dataset consisting of 6685 incident reports and multilabel compatible ML models. Interestingly, Syed et al.^38^ reported that the Linear Support Vector Machine (SVM) was the optimal ML model for their radiation oncology incident report data. This is consistent with our result, as our optimal ML model was the Linear SVR model, which is a regressor derivative of SVM. It is interesting that a regressor model worked better than a classifier model for our classification problem. We believe that it is because of the fact that the regressor models, like Linear SVR, are shown to work well even when the data are sparse and imbalanced like what we had, compared to our classifiers that underperform in such conditions. However, it must be noted that we have only tuned the hyperparameters of the top three models to compare. All other models that we evaluated used default parameter settings. Therefore, it is possible that a model, other than the top three, could potentially outperform our chosen models when the hyperparameters are optimized.

Our work has several limitations, including the inherent imbalance of label distributions in our dataset. As seen from Figures 2, 3, and 4, some class labels are more common than the others, which introduces a skewness in the model training because the model is trained unequally on all the labels. In this work, however, we did not attempt to solve this problem of label imbalance. Although the repercussions of this will not reflect in our model performance because it is representative of the real‐world scenario, more balanced data are needed to improve the model's generalizability. Even then, a model can only be as good as the data themselves and, as mentioned earlier, the free‐text incident descriptions had many human errors. Our NLP processing pipeline was designed to address some of these errors using techniques such as autocorrection but such methods are not perfect. For example, the autocorrection function made about two mistakes for every ten corrections according to a manual examination of a subset of our data. Additionally, in our pipeline, we lose term negations (e.g., “no treatment”) and we did not process abbreviations. While the inclusion of bigrams and trigrams was an attempt to account for the negation loss, most negations were already lost during the stopword removal stage itself. Another major factor that impacts model performance is the subjectivity involved in the manual labelling of these incident reports. We consider the labels assigned by various investigators as our ground truth for model training, but these data can have inaccuracies introduced by the subjectivity of investigating personnel.

It is important to note that the mean TrueLabelIndex score obtained with the contributing factor model (TrueLabelIndex score of 2.66) was inferior to that obtained with the process step and problem type models (TrueLabelIndex scores of 1.48 and 1.73, respectively). There are two likely reasons for this, the first of which is that the contributing factor data element has 25 label options, which is considerably more than that for the process step and problem type (8 and 16, respectively). Indeed, the process step model achieved the highest TrueLabelIndex score and had the lowest number of label options. Secondly, an incident can be labelled with multiple contributing factors, whereas only a single process step and problem type can be assigned per incident. Having more labels to learn, from the same features of the incident descriptions, may reduce the efficiency and accuracy of learning.

Despite these various shortcomings, the final results of applying our optimal models to all three data elements appear very promising. Comparison of the TrueLabelIndex scores obtained with the ML model and the benchmark analytical approach showed significant improvement. Potentially, with deep‐learning techniques, we may be able to improve the performance further in the future. We will incorporate these models into the SaILS investigator interface for each of the data elements considered. In real‐time operation, each model will analyze the reported incident description and provide a ranked list of label options in a dropdown menu for the corresponding data element. According to our results, the most appropriate label would appear, on average, within the first three options, which should improve the usability of the SaILS interface for the investigator. Our NLP‐ML pipeline and our trained, tuned models are available under open‐source licenses on GitHub.^39^

## 5 CONCLUSIONS

We built three different NLP‐ML models (MultiOutputRegressor + Linear SVR) that can generate lists of label recommendations for the process step, problem type, and contributing factors data elements of the Canadian NSIR‐RT incident classification taxonomy for radiation oncology. On average, these models place the most appropriate label within the top three label suggestions. The trained models will be used to generate dropdown menus in our radiation oncology incident learning system (SaILS) to semi‐automate the incident investigation process.

## AUTHOR CONTRIBUTIONS

Felix Mathew: Design of work, data acquisition and analysis, drafting and revising of the manuscript, final approval, and agreement to hold responsibility for the accuracy and integrity of the work. Hui Wang: Design of work, data analysis, revision of the manuscript, final approval, and agreement to hold responsibility for the accuracy and integrity of the work. Logan Montgomery: Data acquisition, revision of the manuscript, final approval, and agreement to hold responsibility for the accuracy and integrity of the work. John Kildea: Conceptualization, revision of the manuscript, final approval, and agreement to hold responsibility for the accuracy and integrity of the work.

## CONFLICT OF INTEREST

The authors have no conflict of interest to declare.
